# Asbestos Induces Reduction of Tumor Immunity

**DOI:** 10.1155/2011/481439

**Published:** 2011-10-04

**Authors:** Naoko Kumagai-Takei, Megumi Maeda, Ying Chen, Hidenori Matsuzaki, Suni Lee, Yasumitsu Nishimura, Junichi Hiratsuka, Takemi Otsuki

**Affiliations:** ^1^Department of Hygiene, Kawasaki Medical School, 577 Matsushima, Kurashiki 701-0192, Japan; ^2^Division of Bioscience, Department of Biofunctional Chemistry, Okayama University Graduate School of Natural Science and Technology, 3-1-1 Tsushima-naka, Okayama 700-8530, Japan; ^3^Division of Pneumoconiosis, School of Public Health, China Medical University, 92 North 2nd, Heping District, Shenyang 110001, China; ^4^Department of Radiation Oncology, Kawasaki Medical School, 577 Matsushima, Kurashiki 701-0192, Japan

## Abstract

Asbestos-related cancers such as malignant mesothelioma and lung cancer are an important issue in the world. There are many conflicts concerning economical considerations and medical evidence for these cancers and much confusion regarding details of the pathological mechanisms of asbestos-induced cancers. For example, there is uncertainty concerning the degree of danger of the iron-absent chrysotile compared with iron-containing crocidolite and amosite. However, regarding bad prognosis of mesothelioma, medical approaches to ensure the recognition of the biological effects of asbestos and the pathological mechanisms of asbestos-induced carcinogenesis, as well as clinical trials to detect the early stage of mesothelioma, should result in better preventions and the cure of these malignancies. We have been investigating the immunological effects of asbestos in relation to the reduction of tumor immunity. In this paper, cellular and molecular approaches to clarify the immunological effects of asbestos are described, and all the findings indicate that the reduction of tumor immunity is caused by asbestos exposure and involvement in asbestos-induced cancers. These investigations may not only allow the clear recognition of the biological effects of asbestos, but also present a novel procedure for early detection of previous asbestos exposure and the presence of mesothelioma as well as the chemoprevention of asbestos-related cancers.

## 1. Introduction

The fact that inhaled asbestos causes malignant mesothelioma and lung cancer is an enormous medical and social problem. Canada's decision to expand asbestos mining and export to developing countries in which asbestos has not been banned is unsettling [1]. People are sometimes influenced by economical forces even though they know many people have suffered from malignant diseases caused by these minerals, and their decisions appear to be made for financial reasons.

In Japan, the asbestos issue erupted in the summer of 2005 [2–4]. Residents were suddenly informed that asbestos, which was used in large amounts from the early 1950s up to the early 1990s in Japan with a maximum usage of approximately 352,000 tons in 1974, caused malignant mesothelioma (MM). Residents that lived near the asbestos handling manufacturer Kubota Corporation, in Amazasaki City, Hyogo Prefecture, developed MM. They had never worked in the asbestos-handling manufacture industry. In addition, medical information regarding MM induced anxiety in the Japanese people, since the prognosis is very poor, and there is no certain way to detect the cancer in the very early stage of the disease. Furthermore, people could not remember being exposed to asbestos 30 to 40 years ago. To reduce the anxieties of the Japanese people, epidemiological analyses regarding the Amagasaki area proceeded, and clinical and basic research was conducted on the biological effects of asbestos and early detection of mesothelioma. It is in this context that the authors became involved in the project “Comprehensive Approach on Asbestos-Related Diseases”, supported by the “Special Coordination Funds for Promoting Science and Technology” (Dr. Takemi Otsuki, Department of Hygiene, Kawasaki Medical School, Kurashiki, Japan) from 2006 to 2010. In this project, a case and clinical specimen registration system was established. A feasibility clinical trial was established and involved a combined trimodality therapy using anticancer chemotherapy with cisplatin and pemetrexed, following by extrapleural pneumonectomy and postoperative radiation therapy for early-stage mesothelioma patients [5, 6]. Furthermore, early detection procedures were developed using serum or pleural effusions to measure soluble mesothelin-related peptide (SMRP) and other markers such as osteopontin, vascular endothelial growth factor (VEGF) and angiopoietin-1 [7–9], as well as procedures for detection of circulating mesothelioma cells and circulating epithelial cells using peripheral blood [10, 11]. 

For the basic research, the project “Comprehensive Approach on Asbestos-Related Diseases” included three subgroups: (1) analyses of cellular and molecular characteristics using mesothelioma cell lines, (2) investigation of asbestos-induced carcinogenesis using an animal model, and (3) study of the immunological effects of silica/asbestos. 

The first subgroup explored novel tumor suppressor gene(s) in mesothelioma cells and found that the serine/threonine-protein kinase (LATS2) gene is inactivated in approximately one-third of mesothelioma cell lines and is a candidate for a novel tumor suppressor in MM [12]. In addition, they found the possibility that the Yes-associated protein (YAP) involved the NF2/Merlin-hippo signaling pathway and that LATS2 may constitutively dephosphorylate and act as an oncogene to bind with the TEAD transcription factor to enhance the cell cycle and resistance to apoptosis [13]. In addition, mesothelioma-specific epigenetic profiles were identified for differential diagnosis with lung adenomatous cancers [14].

The second subgroup confirmed the importance of iron in asbestos-induced carcinogenesis. Findings showed that not only iron-containing crocidolite and amosite, but also chrysotile asbestos caused mesothelioma when these materials were injected into the peritoneal region of a rat. Even individual rats having mesothelioma caused by the injection of iron-absent chrysotile showed numerous depositions of iron in the spleen, liver, and kidney. In addition, adding nitrilotriacetate (NTA) to chrysotile-injected rats induced the acceleration of mesothelioma formation, suggesting the critical participation of iron for asbestos-induced carcinogenesis even for chrysotile. Although the detailed mechanisms of this phenomenon are now being explored, the binding ability of chrysotile to hemoglobin and other proteins and the induced hemolysis is a concern [15–18]. Moreover, the importance of a homozygous deletion of CDKN2A/2B was found in rat mesothelioma with the suggestion that this deletion seems to be fundamental for the development of mesothelioma, since these genes are also known to be homozygously deleted in human mesothelioma [19].

We have performed the third subtheme concerning the “immunological effects of silica/asbestos”. In this paper, we introduce our findings and considerations regarding involvement of reduced tumor immunity caused by asbestos exposure to immunocompetent cells as the basic condition in asbestos-exposed people who may develop MM.

## 2. Immunological Effects of Asbestos

Asbestos comprises a set of six naturally occurring silicate minerals (chrysotile as Serpentine and crocidolite, amosite, actinolite, anthophyllite, and tremolite as Amphibole) exploited commercially for their desirable physical properties. They all have in common their asbestiform structure, possessing long (having more than 1 : 3 aspect ratio, usually approximately 1 : 20) and thin fibrous crystals [20, 21]. Silica (SiO_2_) certainly affects the human immune system, because people exposed to silica not only suffer from respiratory disorders known as silicosis, but also experience complications with autoimmune disorders such as rheumatoid arthritis (known as Caplan's syndrome), systemic sclerosis, systemic lupus erythematosus, and antineutrophil cytoplasmic antibody- (ANCA-) related vasculitis/nephritis [22–27]. We have, therefore, been exploring the mechanisms involved in silica-induced dysregulation of autoimmunity using case peripheral blood specimens. We had found that there is dysregulated expression of the CD95/Fas molecule, which is very important for the survival of self-recognizing T cell clones. Additionally, analyses of Fas and Fas-related molecules in silicosis patients suggested that there are two populations of T cells: one is the long-term surviving populations probably including self-recognizing clones, and the other is a population repeating apoptosis caused by silica and recruiting from the bone marrow [28, 29]. In addition, our recent studies regarding CD4+25+ and forkhead box P3 (FoxP3)+ regulatory T cells (Treg) suggested that (1) silica activates both responder T cells (Tresp) and Treg, (2) Tresp chronically-activated by silica becomes CD4+25+ (and programmed cell death-1 (PD-1) + as an activated cell marker) expressers, (3) Treg activated by silica express higher CD95/Fas and are sensitive to Fas-mediated apoptosis, and (4) after the ongoing progression of these events, the composition of the peripheral CD4+25+ fraction in silicosis patients changes to reflect a loss of Treg and a gain of activated Tresp, and this reduction of Treg function results in activation of autoimmunity in silicosis patients [30–32].

**Figure 1:**
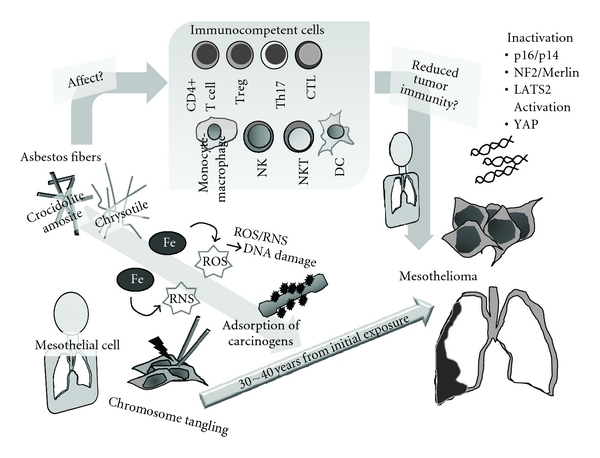
Schematic model showing mechanisms of asbestos-induced carcinogenesis and genomic/epigenetic changes found in mesothelioma cells and the relationship of the immunological effects of asbestos in regard to reduced tumor immunity.

Since silica influences the human immune system, its mineral silicate, an asbestos, may also have an effect. As we considered silica's immunological effects from the complications of silicosis and autoimmune diseases, the most important complication of asbestos-exposed people is the occurrence of malignant disease such as MM and lung cancer. In addition, some epidemiological studies suggested a relationship between asbestos exposure and other cancers of the gastrointestinal tract, larynx, kidney, liver, pancreas, ovary, and hematopoietic systems [33–35]. Thus, if asbestos affects the immune system, a reduction of tumor immunity may result and then make people exposed to asbestos sensitive to the development of malignancies. Of course, asbestos itself possesses carcinogenic activities. As shown in Figure 1, asbestos fibers having iron (or even chrysotile as mentioned above) produce reactive oxygen/nitrogen species (ROS/RNS) causing DNA damage to nearby cells, and fibers are sometimes directly inserted into the cells and injure chromosomes, while retained fibers may adsorb other carcinogens on their surface (known as an asbestos body) [15, 16, 18, 36, 37]. As a result, specific DNA alterations may result, such as inactivation (mostly homozygous deletion) of *p16^INK4a^/p14^ARF^*, *NF2/Merlin,* and *LATS2*, and the activation of *YAP* as mentioned above [12, 13]. However, it is difficult to explain why the development of mesothelioma requires 30 to 40 years, and how asbestos-exposed people possess sensitive features for other cancers. 

We have been considering that asbestos may affect immunocompetent cells such as CD4+ Tresp, Treg, Th17 T cells, CD8+ cytotoxic T cells (CTL), monocyte-macrophage, natural killer (NK) cells, natural killer T (NKT) cells, and dendritic cells (DC). Firstly, to observe the effects of low-dose and continuous exposure to asbestos (we initially chose chrysotile because this is the most frequently used asbestos in Japan, and our investigations suggested it was not carcinogenic), we employed a human adult leukemia/lymphoma virus-1 (HTLV-1) immortalized polyclonal T cell line, MT-2 [38, 39]. In the next part of this paper, analysis of asbestos exposure to the MT-2 cell line is documented.

## 3. Transient and Continuous Exposure to Asbestos on a Human T Cell Line

**Figure 2:**
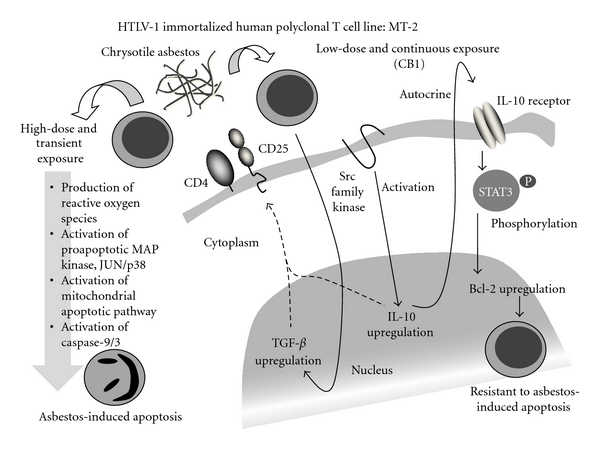
Summarized findings of cellular and molecular events caused by high-dose and transient exposure (left side) and lowdose and continuous exposure (CB1: one of the sublines established) (right side) to chrysotile asbestos using an HTLV-1 immortalized human polyclonal T cell line, MT-2.

Initially, the cellular alteration of MT-2 cells exposed to transient and high-dose chrysotile was observed to compare various published investigations showing the ability of asbestos exposure to induce ROS production and mitochondrial-pathway-dependent apoptosis in normal alveolar epithelial cells and mesothelial cells, which are the target cells of asbestos-induced carcinogenesis. As shown on the left side of Figure 2, transient and relatively high-dose exposure (25–50 *μ*g/mL, not likely to comprise adhesive cells such as alveolar epithelial or mesothelial cells, since we are using suspended cells, and thus, *μ*g/mL was used instead of *μ*g/cm^2^) caused production of ROS as measured by production of *O*_2_^−^ using flow cytometry, phosphorylation of proapoptotic molecules in the mitogen-activated protein kinase (MAPK) pathway such as p38 and c-Jun N-terminal kinase (JNK), release of cytochrome-c from mitochondria to the cytosol, BAX overexpression, cleavage of caspase-9 and -3, and thereafter the appearance of apoptosis [40]. These findings resembled the effects of asbestos on alveolar epithelia and mesothelial cells [41–44].

We then conducted a trial to establish a low-dose and continuous exposure cell line model by adding 5 or 10 *μ*g/mL of chrysotile (doses which cause apoptosis in less than half of cells exposed transiently) to the MT-2 cell culture. After more than eight months exposure with monthly monitoring for the occurrence of apoptosis, and when these cells were re-exposed to fibers one week after being released from continuously exposed chrysotile, an MT-2 subline which showed resistance to chrysotile-induced apoptosis had been established. As shown on the right side of Figure 2, the continuously exposed subline of MT-2 showed activation of Src-family kinase, increased expression and production of interleukin (IL)-10, phosphorylation of signal transducer and activator of transcription 3 (STAT3) with overexpression of BCL-2 (located downstream of STAT3) [45, 46]. In addition, transforming growth factor (TGF)-*β* was upregulated [47, 48]. Actually, we had established three independent continuously exposed sublines to chrysotile B and three other sublines exposed to chrysotile A. The altered gene expression of these six continuously exposed sublines in comparison with the original MT-2 cell line was very similar, and the cellular and molecular characteristics of these cell lines in regard to tumor immunity with the *ex vivo* chrysotile exposure model using freshly isolated peripheral blood CD4+ T cells derived from healthy donors was investigated and confirmed using peripheral blood specimens derived from asbestos-exposed patients such as patients with pleural plaque (PP) or MM.

Chemokine receptor, CXC chemokine receptor (CXCR)3, expression and relation with interferon (IFN)-*γ*.

Using the above-mentioned MT-2 original cell line and the continuously exposed chrysotile sublines, molecules related to tumor immunity were investigated. For example, CXCR3 expression was a focus of investigations, since CXCR3 downregulation in sublines was detected in comparison with the original line using cDNA microarray analysis. It is known that CXCR3 expression and IFN-*γ* production are induced by T-cell activation and lead to the enhancement of antitumor immune function [49, 50]. 

**Figure 3:**
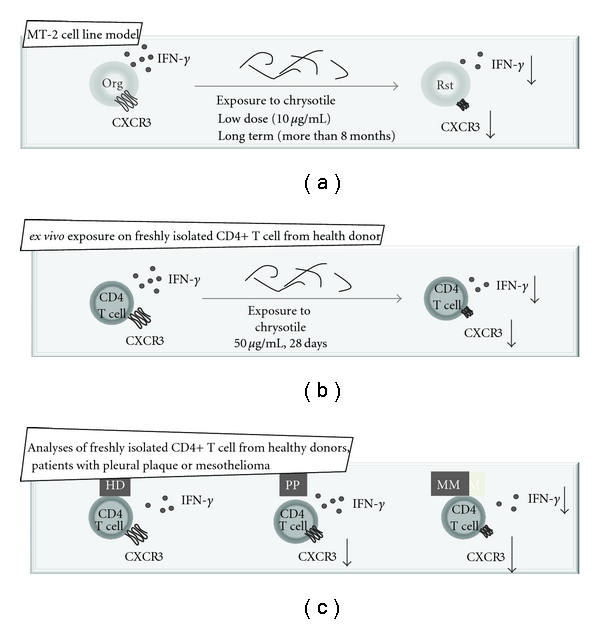
Schematic representation of asbestos-induced reduction of expression of a chemokine receptor, CXCR3, and expression and production of IFN-*γ* using the MT-2 cell line model (Org; MT-2 original cell line, and Rst: sublines exposed continuously to a low-dose of chrysotile), an *ex vivo* exposure model using freshly isolated CD4+ T cells from healthy donors (HD), as well as analyses of freshly isolated CD4+ T cells from healthy donors and patients with pleural plaque (PP) and malignant mesothelioma (MM).

From findings using the MT-2 cell line model, as shown in Figure 3(a), all six continuously exposed sublines showed reduced CXCR3 expression on their surface and mRNA expression levels with reduced production and expression of IFN-*γ*. Production of the Th1-type CXCR3 ligand CXCL10/IP10 was also significantly reduced in all six continuously exposed sublines when compared with the original line. In addition, another Th1-type chemokine, CCL4/MIP-1*β* mRNA, was also expressed at low levels in all six sublines compared with the MT-2 original line as previously reported. However, CCR5, the Th1-type receptor for CCL4/MIP-1*β*, was not reduced significantly through mRNA expression in MT-2Rsts cells. These results indicated that continuous exposure of MT-2 original cells to asbestos altered the expression of Th1-related chemokines (CXCL10/IP10 and CCL4/MIP-1*β*) and chemokine receptors (CXCR3) [51]. 

Thereafter, we tried to determine whether freshly isolated human peripheral CD4+ T cells show a similar alteration *ex vivo *when proliferation is maintained by IL-2-containing medium in the presence of chrysotile as shown in Figure 3(b). After 40 days of coculture supplemented with IL-2 in the presence or absence of chrysotile, cell surface CXCR3 expression decreased in a dose-dependent manner. Thus, we examined cell surface expression of CXCR3 and CCR5 in CD4+ T cells derived from six healthy donors, since both receptors are preferentially expressed in Th1/effector T cells. The expression of CXCR3 was significantly reduced following exposure to 10 *μ*g/mL of chrysotile for 28 days although this difference seemed to depend on one case in which the expression decreased remarkably. Even if the culture conditions for the CD4+ T cells was limited to a period of around four weeks, four of the six healthy donors showed a decrease of CXCR3 expression to various degrees, and it might be concluded that asbestos exposure potentiates reduction of CXCR3 expression in CD4+ T cells. On the other hand, the expression of CCR5 varied among all healthy donors, and there were no significant changes after seven and 28 days of coculture with chrysotile, as shown previously by the cell line model. These results indicated that CXCR3 expression might be specifically reduced by asbestos exposure. In addition, these experiments revealed decreased IFN-*γ* expression and production when CD4+ T cells from healthy donors were cultured with chrysotile for 28 days [52]. 

Finally, analyses of changes in surface CXCR3 expression on freshly isolated CD4+ T cells from asbestos-exposed patients such as PP or MM were compared with those from health donors. In addition, IFN-*γ* expression of CD4+ T cells from these patients and healthy donors was measured with stimulation using anti-CD3/CD28 antibodies with IL-2. As summarized in Figure 3(c), CXCR3 expression was reduced in CD4+ T cells from asbestos patients. A comparison of PP and MM patients showed that the expression level of CXCR3 on CD4+ T cells from MM was decreased although the difference was not statistically significant. Moreover, IFN-*γ* expression was only reduced in stimulated CD4+ T cells from MM patients, not in those from PP patients [52]. 

With the findings that CD4+CXCR3+ T cells in lymphocytes from MMs showed a tendency for an inverse correlation with CXCR3's ligand, CXCL10/IP10 in plasma, our results indicate a reduction of tumor immune function in asbestos-exposed patients and suggest that CXCR3, IFN-*γ*, and CXCL10/IP10 may be candidates to detect and monitor disease status.

## 4. Alteration of NK Cells and Others

**Figure 4:**
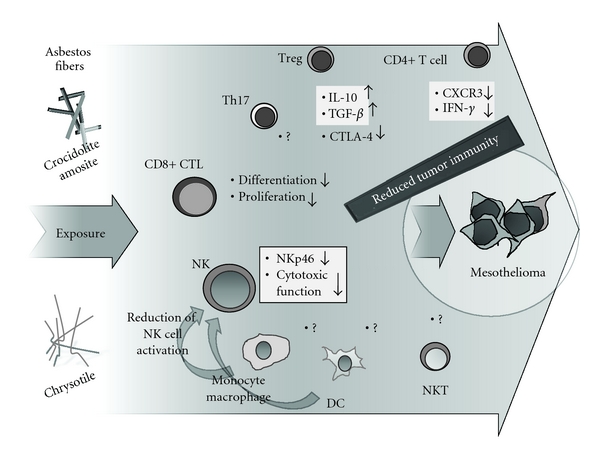
Schematic representation of findings showing asbestos-induced reduction of tumor immunity on CD4+ T cells, CD4+25+FoxP3+ regulatory T cells (Treg), T helper (Th)17, CD8+ cytotoxic T cells (CTL), natural killer (NK) cells, monocyte-macrophage, dendritic cells (DC), and natural killer T cells (NKT).

As shown in Figure 4, the effects of asbestos on other immunocompetent cells such as Treg, CD8+ CTL, and NK cells were investigated. As mentioned above with the MT-2 cell line model, sublines continuously exposed to chrysotile showed overproduction of IL-10 and TGF-*β*. It is well known that these cytokines are a typical soluble factor produced from Treg to function with a suppressive effect on activated responder T cells. On the other hand, it is also reported that MT-2 cells possess a Treg function, since cells express CD4 and CD25 with nuclear expression of FoxP3. Taken together, continuous exposure to chrysotile produces a stronger Treg function, at least with the capacity to produce soluble functional factors (i.e., IL-10 and TGF-*β*) [47, 48]. At present, we have been studying alteration of Treg function using the MT-2 cell line model, and preliminary findings indicate asbestos may enhance Treg function.

In regard to tumor immunity, CD8+ CTL and NK cells are very important players, since they directly kill tumor cells even when individually restricted with major histocompatibility complexes. Investigations have just started with CD8+ CTL, but in *ex vivo* experimental conditions designed to produce CD8+ CTL proliferation and differentiation using freshly isolated peripheral blood mononuclear cells from healthy donors, the addition of asbestos seems to result in reduced proliferation and differentiation of CTL. Although detailed analyses concerning the roles of cytokines surrounding CTL differentiation are being performed, our ongoing studies suggest that asbestos reduces CTL activities.

Regarding NK cells, cellular and molecular analyses have been conducted using a human NK cell line, YT-A1, exposed continuously to asbestos in an *ex vivo* exposure model using freshly isolated NK cells from health donors, as well as asbestos-exposed patients such as PP and MM. 

Focusing on the NK cell-activating receptors, including NKG2D (also known as KLRK1 (killer cell lectin-like receptor subfamily K, member 1), klr and CD314, binding to a diverse family of ligands that include MHC class I chain-related A and B proteins and UL-16 binding proteins, where ligand-receptor interactions can result in the activation of NK and T cells), 2B4 (also known as NAIL; Nmrk; NKR2B4; SLAMF4 and CD244, mediate nonmajor histocompatibility complex (MHC) restricted killing), and NKp46 (also known as NCR1 (natural cytotoxicity triggering receptor 1), LY94 and CD335, constituting a natural cytotoxic receptor family with NKp44 and NKp30, and being important in killing tumor cells and dendritic cells), the YT-A1 human NK cell line exposed continuously to chrysotile asbestos revealed reduced expression of NKG2D and 2B4 [53]. The reduced phosphorylation of extracellular signal-regulated kinase (ERK) and subsequent reduction of degranulation of perforin and granzyme B resulting from reduced cytotoxicity were observed in this cell line model [54]. Similar to the cell line model, the *ex vivo* exposure model using freshly isolated NK cells from healthy donors revealed a reduction of NKp46 expression. Furthermore, freshly isolated NK cells from MM patients showed reduced killing function compared with those from healthy donors and revealed a lower expression of NKp46 [53]. Moreover, the expression level of NKp46, but not NKG2D or 2B4, and the cytotoxic activity of individual freshly isolated NK cells from health donors and MM patients clearly showed a reverse correlation, indicating that the target molecule of asbestos-induced dysfunction of NK cells is NKp46 [54]. Although further analyses are required regarding the interaction between asbestos-exposed NK cells and other immunocompetent cells such as dendritic cells, monocytes, and macrophages, molecular mechanisms to reduce NKp46 expression and other aspects need to be explored, and surface NKp46 expression levels may be the candidate to monitor the level of tumor immunity in asbestos-exposed patients [55].

Further investigations are needed to examine the effects of asbestos exposure on other types of immunocompetent cells such as Th17 dendritic cells, NKT, and the monocyte-macrophage lineage, and to investigate why asbestos seems to reduce tumor immunity in the total network of the immunological surveillance system.

In addition, although we have mainly analyzed the effects of chrysotile asbestos on the human immune system, differences and similarities between the different types of fibers should also be investigated.

## 5. Conclusion

We have been investigating the effects of asbestos exposure on the human immune system in regard to tumor immunity and found that people exposed to asbestos possess reduced tumor immunity, making them sensitive to cancer development. Although these studies may contribute to the clear recognition of the biological effects of asbestos, the variety of alterations in immunocompetent cells may be the factor that allows detection of previous asbestos exposure and the occurrence of cancer in people that live or have lived near asbestos-handling manufacturers. Furthermore, to recover tumor immunity using physiologically active substances in foods or derived from plants may be an effective method for the chemical prevention of asbestos-induced cancers.
